# An examination of perseverative errors and cognitive flexibility in autism

**DOI:** 10.1371/journal.pone.0223160

**Published:** 2021-01-13

**Authors:** Oriane Landry, Peter Mitchell

**Affiliations:** School of Psychology, University of Nottingham, Nottingham, United Kingdom; Central European University, HUNGARY

## Abstract

Perseveration is a well-replicated finding in autism. The aim of this study was to examine how the context of the task influences performance with respect to this phenomenon. We randomly assigned 137 children aged 6–12 with and without autism to complete a modified card-sorting task under one of two conditions: Children were either told the sorting rules on each trial (Explicit), or were given feedback to formulate the rules themselves (Implicit). While performance was enhanced on the Explicit condition for participants without autism, the participants with autism were disadvantaged by this manipulation. In contrast, there were few differences in performance between groups on the Implicit condition. Exploratory analyses were used to examine this unexpected result; increased autism symptomology was associated with poorer performance.

## Introduction

Errors committed by participants with autism on executive function tasks such as the Wisconsin Card Sort Test (WCST;[1]) are said to reflect cognitive inflexibility; participants become *stuck in set*, such that once a response set or strategy is established participants cannot abandon that for a new strategy and instead persevere with the old strategy despite the fact it is no longer effective. The aim of the following study was to further investigate this phenomenon in the context of developmental psychology, by examining performance relative to the typical developmental trajectory of cognitive flexibility. Namely, while the perseverative errors committed by individuals with autism on the WCST resemble the response patterns of adults with frontal lobe damage, they also resemble the response patterns of 3-year-old typically developing children[2, 3]. We experimentally manipulated the WCST, drawing also from its pre-school analogue task, the Dimensional Change Card Sort (DCCS) [4], administered under two sets of conditions, in order to further understanding of perseverative behaviour in autism.

### Cognitive flexibility and executive function in autism

A range of assertions have been made with respect to whether and which aspects of executive functions are intact or impaired in persons with autism, depending on the tasks used to measure particular sub-skills [5–11]. The WCST [1] is one of the most widely used tests of executive function, broadly defined, with a range of outcome measures representing different aspects of performance [2]. The task consists of four target cards that differ on three dimensions (colour, shape, and number). Participants are given test cards that vary along the same dimensions and are asked to place the test card with the target card that is the best match. Participants are told only whether their choice was correct or incorrect, and must then infer the sorting rule according to this feedback. After ten correct responses, the sorting rule is changed without notice to the participant, who must then deduce the new rule. This test is classed as a test of “cognitive flexibility” or “attentional set-shifting” because of the changing rules. It has been used extensively with persons with autism, revealing they complete fewer categories and commit more perseverative errors than matched control groups [12–19] (but see [20–22] for notable exceptions). Due to the complex nature of the task, however, the source of error is unclear.

On the WCST, three types of error are often measured: failure to maintain set (FMS), perseverative error, and non-perseverative / random error. An FMS error occurs when the participant changes response strategies despite the rule remaining the same. This type of error is regarded as attentional [23]. Perseverative error occurs when the participant continues with the same response strategy following a rule switch. This type of error is regarded as a failure to inhibit a prepotent response. Non-perseverative errors are generally considered to be random. Both perseverative and non-perseverative errors are considered to reflect executive dysfunction [23]. Non-perseverative error rates are not reported as frequently as perseverative error rates, but it seems persons with autism commit both types of error more often than comparison groups [18, 24], while the proportion of total errors classified as perseverative does not differ between groups. In a recent meta-analysis, Landry and Al-Taie [2] concluded that perseverative, non-perseverative, and FMS errors occur more often in individuals with autism, and that there is no evidence that individuals with autism perform better on the WCST when the task is administered on a computer.

The WCST is a complex task, and it may be an oversimplification to state that flexibility underlies errors committed by individuals with autism on the task. For example, the Intra-dimensional/Extra-dimensional shift test (ID/ED) test contains nine levels of increasing complexity (see Hughes, Russell, & Robbins [5] for complete description). On this task, children, adolescents, and adults with autism demonstrated flexibility on the first seven levels, which included simple reversals, compound reversals, and intra-dimensional shifts and reversals, and were inconsistently reported impaired on the extra-dimensional shift, when the requirement was to attend to the previously irrelevant dimension [5, 6]. Like the WCST, the ID/ED task requires participants to figure out the rule on the basis of feedback, when the rule changes after a certain criterion of correct responses is attained. The ID/ED task therefore also provides evidence against the argument that participants with autism are insensitive to feedback, as participants were successful in switching response rules following feedback. The ID/ED task is different from a card-sorting task in that participants learn to respond to one of a pair of stimuli, rather than sort stimuli into categories. Participants with autism demonstrated flexibility on the ID/ED conditions up to and including novel exemplars and reversal within the same basic higher-order rule, when discrimination is based on shape. Participants with autism from ages 7 through 47 ran into difficulty when the higher-order rule changed (ignore shape) and a previously irrelevant dimension of the stimuli became the basis for the rule [5, 11].

In summary, participants with autism are prone to a specifically “stuck-in-set perseveration” [5] that is not accounted for by insensitivity to feedback. Another task in which higher-order rules change and the previously irrelevant dimension of the stimuli becomes the basis for the rule is the DCCS. The DCCS [25] was created as an analogue of the WCST for young children, and contains only two targets and two potential sorting strategies. Further, on the DCCS, children are told how to sort the cards on every trial and very explicitly told when the sorting strategy changes. Despite the explicit provision of sorting rules, 3-year-olds are perseverative and inflexible. When 5-year-olds are provided with the sorting rules, however, they very reliably switch sorting strategies on the DCCS [26], yet commit higher rates of perseverative and non-perseverative errors on the WCST compared with 12 year olds [27]. Perseveration on the DCCS and perseveration on the WCST are thus developmentally different forms of cognitive inflexibility. To date, limited research has examined perseverative behaviour in autism within this context.

Children with autism demonstrated less flexible performance on the DCCS relative to typically developing children on manual card versions [28] and computerized versions [29, 30]. Preliminary evidence suggested children with autism performed similarly to verbal mental age matched typically developing children on the DCCS [31]. Data reported by Zelazo, Jacques, Burack, & Frye [32] also suggests simple switching behaviour is verbal mental age appropriate in autism. Zelazo et al. [32] tested participants with autism on two tasks designed to assess cognitive flexibility in preschool aged children and provided participants with autism a composite score reflecting performance across the two tasks, one of which was the DCCS. Scores could range from 0–4 with a maximum score of 2 on each task indicating successful switching; individuals scoring 4 thus exhibited switching on both tasks, and individuals scoring 3 must have exhibited switching on one task but not the other. An examination of their data [32], indicates that participants who were inflexible also predominantly had lower verbal mental ages, many equivalent to 3 or 4 years old. Thus, on the DCCS, participants with autism were capable of a level of cognitive flexibility that was commensurate with their developmental level.

### Current study

There are two key differences between the DCCS and the WCST. First, they differ in the number of targets and potential sorting rules. Second, they differ in the instructions and feedback given to participants. For the present investigation, we experimentally manipulated both of these factors. The DCCS contains two target cards and allows two potential conflicting sorting rules. The WCST contains four target cards and three potential conflicting sorting rules. We bridged this gap by creating a card-sorting task with three levels. The first level, like the DCCS, contains two target cards and two conflicting rules. The second level contains three target cards and three conflicting rules. The third level, like the WCST, contains four target cards and three conflicting rules. Unlike the adult WCST, we removed all ambiguous cards—cards that can simultaneously match on more than one dimension. For example, a test card with two blue triangles matches the target of four blue triangles both on colour and shape. This increases the difficulty of the task as participants may receive correct feedback that is ambiguous with respect to which rule is correct, colour or shape. We did not include any such ambiguous cards; a given card could only match one dimension at a time. We administered the task to children in one of two conditions. In the Explicit condition, like the DCCS, children were explicitly told on each trial which rule to use to sort the cards. In the Implicit condition, like the WCST, children were told that they had to figure out which rule to use on the basis of feedback. We tested the prediction that the Explicit condition would be easier than the Implicit condition for all children. Further, we tested the prediction that children with autism would perform differently to children without autism, when matched for developmental level.

## Method

### Participants

Participants included 42 children with prior autism diagnoses and 95 children without autism (80 typically developing and 15 children with developmental delay), ages 6–15. Children were recruited from mainstream public and special education schools in England and Wales. The research was approved by the School of Psychology, University of Nottingham Research Ethics Board. The research was also approved by all participating schools. Written consent was obtained from parents, while participants provided verbal assent. The children with autism were recruited from special education schools and all had received a formal clinical diagnosis prior to school entry according to DSM-IV [33] or ICD-10 [34] criteria, and as a result had a statement of Special Educational Needs for an Autism Spectrum Disorder. Diagnostic criteria was confirmed using an observational checklist based on the DSM-IV, and current symptomology was quantified using the Social Communication Questionnaire (SCQ; [35]). No participants (with or without autism) had a diagnosis of ADHD according to parent and teacher report. All 95 children without autism completed the experimental task. Of this group, 50 were selected as best potential developmental matches for the participants with autism (including 35 typically developing children with chronological age distributions matched to the mental age distribution of the participants with autism and all 15 children with developmental delay) and completed the Weschler Abbreviated Scale of Intelligence (WASI [36]) and the SCQ [35].

Parents completed the SCQ by mail with a 62% return rate; parents of children with autism completed the Current Form to give a metric of current symptomology, while parents of children without autism completed the Lifetime Form to screen for autism symptoms and give a comparable lifetime metric of sub-clinical symptomology. The SCQ is a 40-item questionnaire that parents can complete without supervision, responding “yes” or “no” to a series of statements about their child. The items on the SCQ parallel the Autism Diagnostic Interview–Revised [37]. The Lifetime Form asks if the child *ever* exhibited symptoms, with many questions focused on the critical period between ages 4 and 5, while the Current Form asks if the child has exhibited the same symptoms in the past three months. Higher scores indicate the presence of more symptoms of autism. The recommended cut-off score for the Lifetime Form is 15, which gives a minimum of false negatives, while a cut-off score of 22 gives a minimum of false positives. Discriminative validity is reported to be .86 overall, but is better for individuals with average IQ and low IQ (.90) than for very low IQ (.79).

Four participants with autism were excluded because IQ testing was incomplete. Five participants without autism were excluded for having SCQ scores ≥ 22. Thus, 38 children with autism and 45 children without autism (35 typically developing and 10 developmental delay) were included in the analyses. Mann-Whitney U test was used to check for differences in WASI raw performance between groups. Groups were matched on performance subscales of the WASI (Block–*U* = 780, *p* = .49, Matrix–*U* = 775.5, *p* = .69) but differed significantly on verbal subscales (Vocab–*U* = 578.5, *p* = .011, Similarities–*U* = 542, *p* = .009). Age, IQ, WASI scores, mental age estimates, and SCQ scores are provided in Table 1. Participants were randomly assigned to the Explicit or Implicit conditions and there were no significant differences between conditions or interactions between condition and group on any of these measures.

**Table 1:** SCQ scores, WASI raw scores, IQ, Age, and mental age estimates for participants with and without autism. No differences were found between conditions. Cohen’s d values are provided for the main effect of group.

		Non-autism				autism				
		(n = 45)				(n = 38)				
		Mean	SE	Min	Max	Mean	SE	Min	Max	Cohen’s *d*
Social Communication Questionnaire^†^*	Implicit	6.69	1.60	1	20	17.46	1.60	11	27	
	Explicit	7.13	1.49	2	21	20.4	1.82	12	32	
	Combined	6.92	1.01			18.73	1.27			2.05
WASI Vocabulary*	Implicit	25.5	2.24	8	46	19.87	2.52	1	47	
	Explicit	23.94	2.52	0	42	16.9	2.52	0	37	
	Combined	24.37	1.53			18.38	1.95			0.54
WASI Blocks	Implicit	10.75	3.09	0	44	19.18	3.48	0	65	
	Explicit	9.26	3.48	1	19	15.1	3.48	0	65	
	Combined	9.89	1.34			17.14	3.20			0.47
WASI Similarities*	Implicit	14.67	1.67	1	29	11.06	1.88	0	31	
	Explicit	15.32	1.88	3	27	9.82	1.88	0	27	
	Combined	14.95	1.08			10.43	1.47			0.55
WASI Matrix Reasoning	Implicit	13.33	1.64	0	27	13.34	1.84	1	29	
	Explicit	12.74	1.84	2	22	12.16	1.84	2	27	
	Combined	13.07	1.13			12.75	1.38			0.04
VIQ*	Implicit	88.2	2.93	55	113	64.95	3.36	55	99	
	Explicit	84.4	3.27	50	107	65.11	3.36	53	88	
	Combined	86.51	2.41			65.02	1.98			1.5
PIQ*	Implicit	89.04	3.72	53	124	77.16	4.27	55	126	
	Explicit	86.8	4.16	61	109	76.37	4.27	54	120	
	Combined	88.04	2.63			76.76	3.12			0.61
Full Scale IQ*	Implicit	87.56	3.17	50	115	68.37	3.64	52	108	
	Explicit	84.25	3.55	55	104	68.16	3.64	50	101	
	Combined	86.09	2.54			68.26	2.27			1.14
Chronological Age*	Implicit	9.6	0.44	6.5	15.42	13.06	0.5	9.92	16.33	
	Explicit	9.4	0.49	6.5	13.75	12.21	0.5	8.92	15.5	
	Combined	9.51	0.34			12.64	0.32			1.45
Verbal Mental Age Estimate	Implicit	8.19	0.35	5.33	12.25	8.46	0.4	5.96	13.53	
	Explicit	7.78	0.39	5.27	11.15	7.91	0.4	5.08	11.32	
	Combined	8.01	0.24			8.19	0.30			0.1
Performance Mental Age Estimate*	Implicit	8.23	0.46	5.4	12.3	10.09	0.53	6.27	15.86	
	Explicit	7.98	0.51	5.33	10.86	9.35	0.53	6.18	15.79	
	Combined	8.12	0.23			9.72	0.48			0.68

^†^62% of parents returned the SCQ.

*Main effect of group p < .05. No effects of condition or interactions between group and condition were found for any variables.

### Stimuli

**Fig 1:**
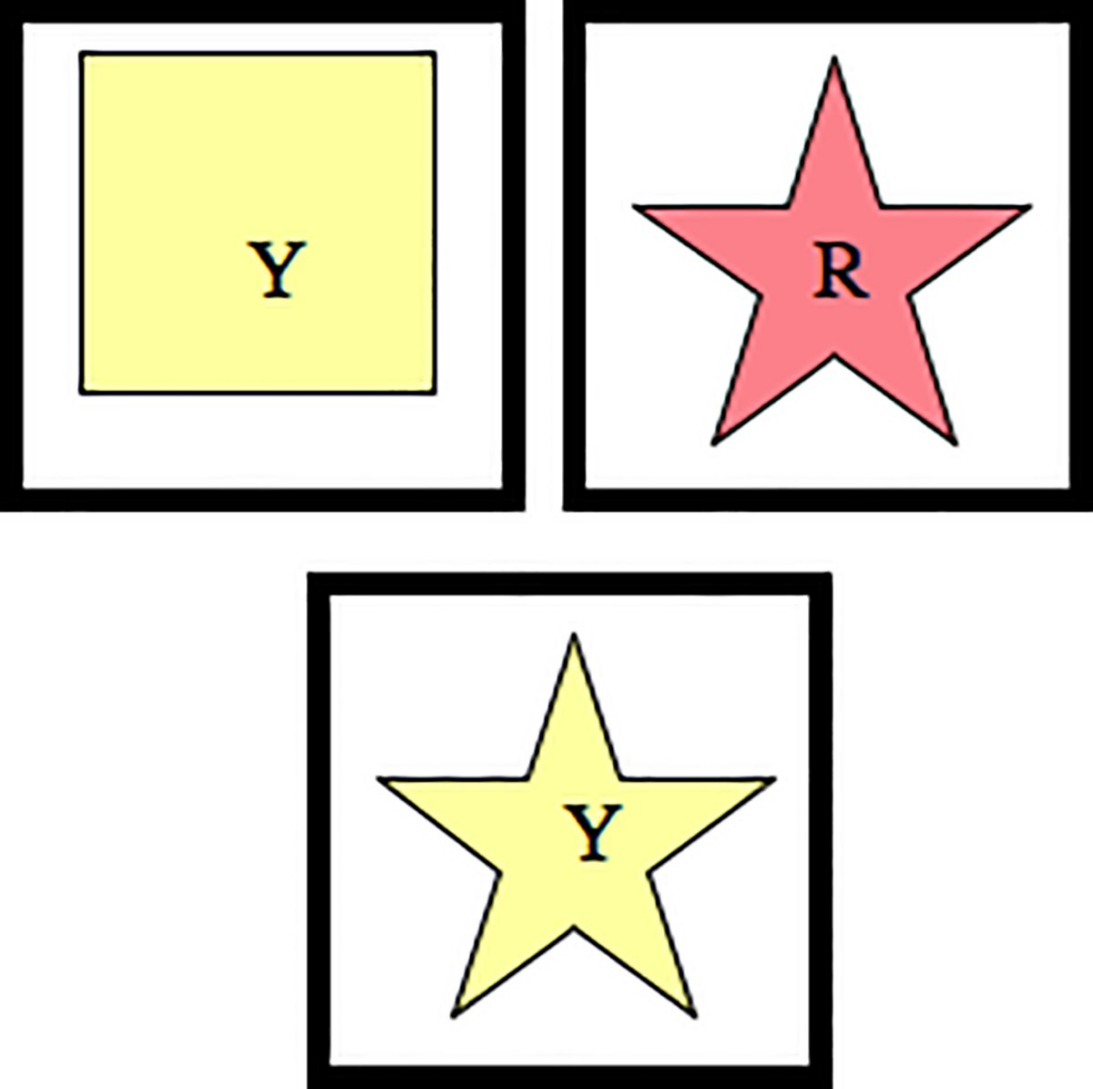
Example of two-target condition. The test card, a yellow star, is matched to the yellow square when the sorting rule is colour, and to the red star when the sorting rule is shape.

**Fig 2:**
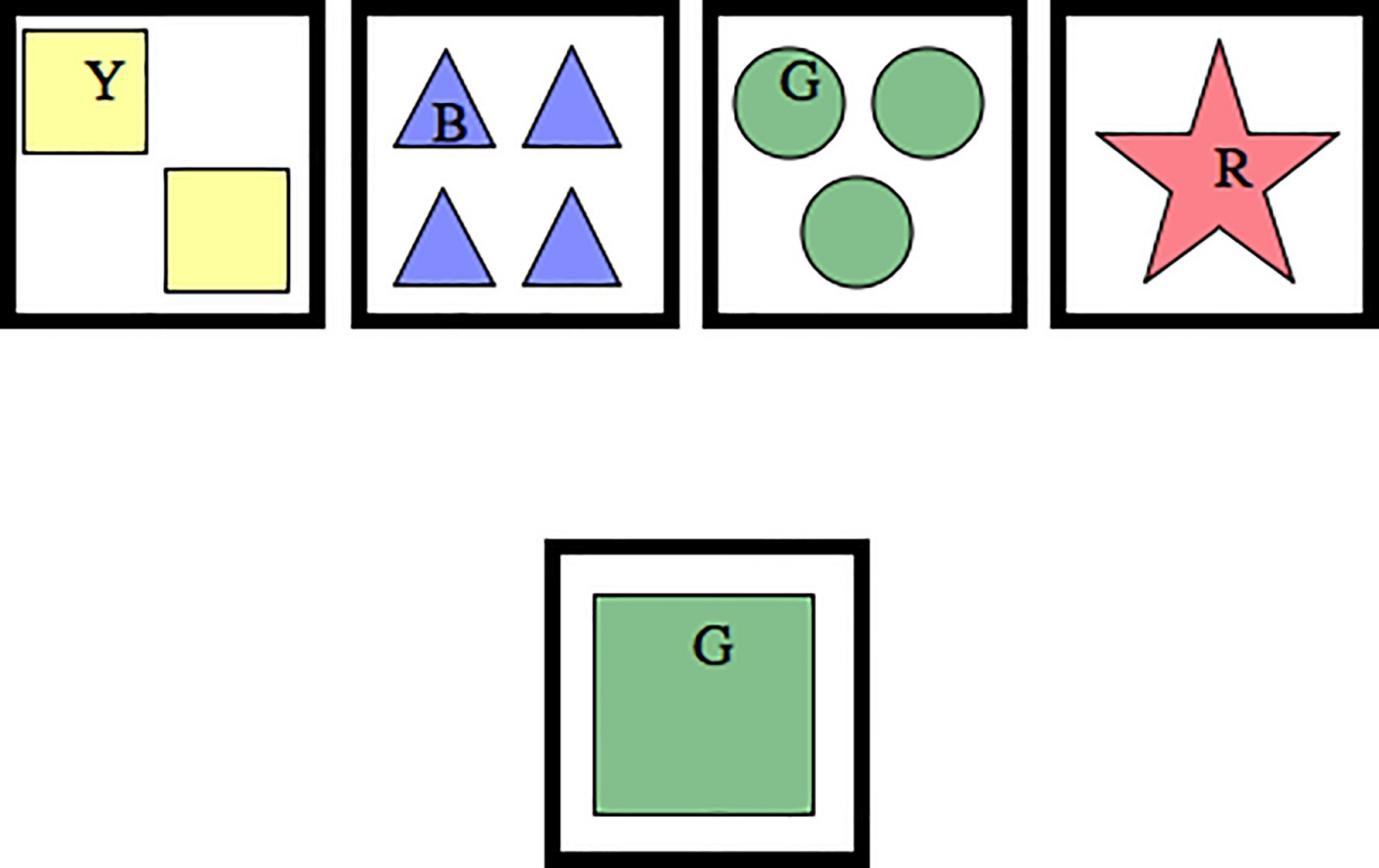
Example of three and four-target condition. Both the three and four-target levels included three possible sorting rules: colour, shape, or number. The three-target condition contained the one red star, two yellow squares, and three green circles. The four-target condition contained these same three targets plus the four blue triangles. The one green square test card can be matched to the two yellow squares when the rule is shape, to the three green circles when the rule is colour, and to the one red star when the rule is number.

The target stimuli and test cards were printed on A6 laminated card stock using an ink-jet printer. The target cards were affixed to trays placed in front of the participants on a table. The target cards for the two-target level included a red star and a yellow square (Fig 1). The target cards for the three-target level included one red star, two yellow squares, and three green circles (Fig 2). To these, four blue triangles were added for the four-target level (Fig 2). A complete list of test cards is given in S1 File.

### Procedure

The participants were randomly assigned to either the Explicit or Implicit condition. All testing occurred in the participants’ schools (in a resource room or library). The participants were introduced to the task as a special card game and given a pre-test to ensure they could identify all four colours, shapes, and numbers using the four-target cards (point to the ____). The four-target cards were removed, the two-target cards were introduced and participants were told how to play the game. The complete script of instructions is provided in S2 File. The test cards were presented to participants one at a time, and participants placed the cards face down in front of the appropriate target card. The test cards could be matched to the target cards on colour, shape, or number. For example, a test card featuring a single green square can be matched to the two yellow squares if the rule is “match to shape,” to the three green circles if the rule is “match to colour,” or to the single red star if the rule is “match to number.” All participants started with the two-target level. Rules changed after participants made 10 consecutive correct responses. If participants were unable to achieve 10 consecutive correct responses in 24 trials, the task was terminated, and the number of successfully completed sets was recorded. Participants completed a maximum of 8 sets, using each sorting rule once per level. The presentation order of the sorting rules was counterbalanced for the two-target level, and randomly generated for the three and four-target levels. Efforts were made to match the intonation and length of the instruction given to the participants in the two conditions. In the Explicit condition, participants were told: “put this with the same _____” before placing each test card. In the Implicit condition participants were told “yes that is right / no that is wrong” after placing each test card. If participants hesitated and looked to the experimenter for guidance, they were prompted, “where does this one go?” When the participants had completed the task, or the task was terminated, they were praised for their performance and debriefed.

## Results

### Data analyses

Testing yielded 5 dependent measures of performance on the two conditions of the task. These included (a) number of sets completed, with possible scores between 0 and 8, (b) number of trials to complete first set, (c) average number of trials per set, (d) number of errors, and (e) average run length, meaning the number of consecutive trials using the same rule regardless of whether correct. Participants were rank-ordered on each of the five dependent measures and those ranks were summed to create a composite score entitled *simple performance*. For the purposes of rank ordering, average run length was recalculated as the absolute value of 10 minus the average run length, such that better performance indicated runs closer to 10 and worse performance indicated runs deviating from 10, high or low. For the ranked variables, higher ranks indicated better performance, thus higher summed ranks indicated better overall performance.

Errors were also broken down into *perseverative*, committing the same error twice, *failure to maintain set* (FMS), committing an error following a string of two or more correct responses, and *random*, which included all other errors not classified as perseverative or FMS. Where appropriate, we used parametric statistical tests. Measures on ordinal scales were analysed using non-parametric tests (Spearman correlations & Mann-Whiney U tests).

A large sample of typically developing children were tested on the experimental task from which participants were selected to developmentally match the participants with autism on the basis of WASI performance as estimates of cognitive developmental attainment. We first examined performance on our task developmentally in our typically developing sample, and then examined performance in autism relative to the comparison sample. Finally, we conducted exploratory analyses to shed light on the performance of children with autism.

### Developmental analysis with typically developing sample

Initially we examined performance of the 80 typically developing children on the task. The participants were grouped according to age (6–8 year olds, *n* = 38, & 9–11 year olds, *n* = 42), and simple performance was analyzed using a 2 (age group) x 2 (condition) ANOVA. A significant main effect of condition, *F* (1,75) = 140.00, *p* < .001, partial *η*^*2*^ = .651, confirmed that performance was better in the Explicit condition (*M* = 276.16, *SEM* = 9.23) than the Implicit condition (*M* = 125.53, *SEM* = 9.31). There were no effects of age group.

A Pearson correlation was used to examine age-related improvements in simple performance. As these correlation analyses were exploratory, the p-values presented were not corrected for multiple comparisons. There was no evidence of association between age and simple performance in the Explicit condition, *r*(38) = -.03, but there was a moderate positive association in the Implicit condition, *r*(38) = .41, *p* = .009. Spearman correlations between age and each of the individual dependent measures were calculated separately for each condition. In the Explicit condition, age negatively correlated with average trials per set, *ρ*(38) = -.34, *p* = .03, and positively correlated with average run length, *ρ*(38) = .40, p = .01. In the Implicit condition, age positively correlated with the number of trials to complete the first set, *ρ*(38) = .36, *p* = .022, the proportion of errors classified as FMS, *ρ*(37) = .42, *p* = .007, but negatively correlated with the proportion of random errors, *ρ*(37) = -.35, *p* = .028.

There were too few errors committed to statistically analyze by type. In the Explicit condition, younger children committed an average of 2.8 errors and older children committed an average of 0.7 errors; for both age groups the errors were predominantly FMS errors. In the Implicit condition (mean total errors 9.1 & 8.7), younger children committed disproportionately more random errors than perseverative or FMS errors, whereas older children committed disproportionately fewer random errors than perseverative or FMS (Table 2). The majority of children in the Explicit condition (all except one younger child) were at ceiling in sets completed and trials to first set. The number of children achieving ceiling performance in the Implicit condition was much lower, with 30% of younger children and 55% of older children completing all eight sets. Thus, the lack of age-related improvement seen in the Explicit condition is due to near ceiling performance achieved by even the youngest participants, confirming that indeed the Explicit condition is easier.

**Table 2:** Error breakdown among typically developing sample. Errors were classified as perseverative, failure to maintain set (FMS), or random. Proportions are based on a very small number of errors committed and thus not analyzed and are included only for descriptive purposes. Proportions were calculated for each participant before aggregation, and thus do not sum to 1.0 (100%).

Condition	Age Group	Type of Error	Mean Proportion	SE
Implicit	Younger	Perseverative	.271	.043
		FMS	.225	.076
		Random	.455	.056
	Older	Perseverative	.321	.044
		FMS	.351	.078
		Random	.275	.057
Explicit	Younger	Perseverative	.094	.045
		FMS	.471	.080
		Random	.046	.059
	Older	Perseverative	.062	.042
		FMS	.271	.074
		Random	.143	.055

### Autism vs. comparison group

**Fig 3:**
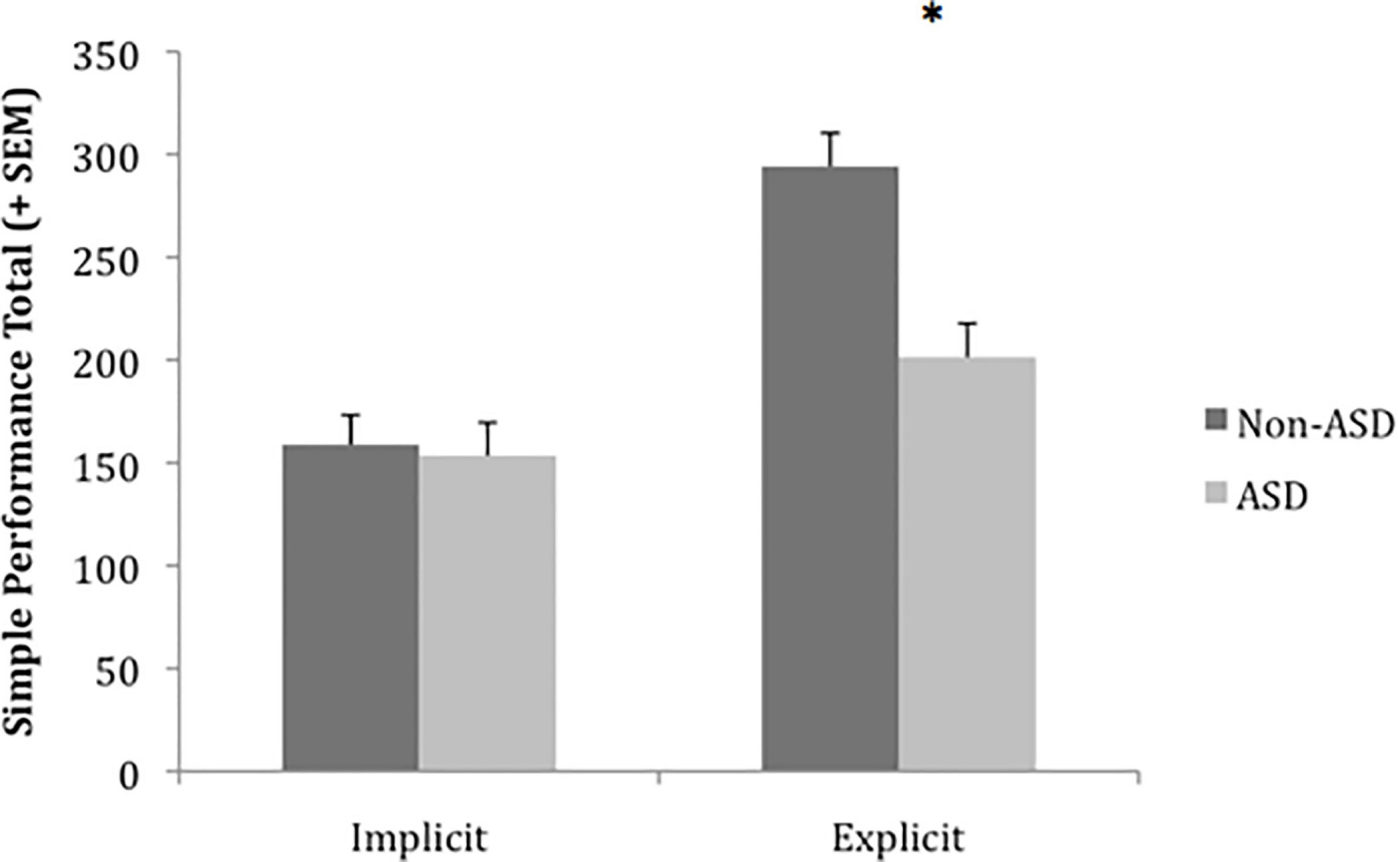
Simple performance composite scores by group and condition with verbal subtest scores covaried. A significant interaction was found. Simple effects tests confirmed a significant effect of group in the Explicit condition but there was no evidence of difference between groups in the Implicit condition.

Simple performance was analyzed using a 2 (group) x 2 (condition) ANCOVA with WASI vocabulary and similarities raw scores as covariates. A significant main effect of group, *F*(1,75) = 9.11, *p* = .003, partial η^2^ = .108, confirmed better overall performance by children without autism. A significant main effect of condition, *F*(1,75) = 34.06, *p* < .001, partial η^2^ = .312, confirmed better overall performance in the Explicit condition. A significant interaction, *F*(1,75) = 7.84, *p* = .007, partial η^2^ = .095, showed that while children in both groups performed better in the Explicit condition, this effect was considerably stronger among children without autism (Fig 3). Further, simple effects tests demonstrated that while there was a significant effect of group in the Explicit condition, *F*(1,75) = 15.89, *p* < .001, partial η^2^ = .175, there was no evidence for difference in overall performance of the two groups in the Implicit condition (*p*>.8).

### Exploratory follow-up analyses

While the hypothesized predictions of a main effect of group and main effect of condition were supported, the pattern of data evident in the interaction inspired further exploratory analyses. These analyses were meant to better understand the performance of children with autism, given the concurrent finding that among a typically developing sample, the Explicit condition is easier, more errors were committed in the Implicit condition, and the type of errors would be expected to differ by condition. In the Explicit condition, errors would be expected to be predominantly of the FMS type, whereas in the Implicit condition, error patterns are more difficult to predict. We performed exploratory analyses on the different metrics of performance, as well as on error patterns.

First, we examined the raw scores on the four non-error metrics that were used to create the simple performance scores. Table 3 provides mean scores for each of these recorded aspects of performance. The data were not normally distributed, and Mann-Whitney U tests were used to test for differences between groups within each condition separately and a Bonferroni correction applied for multiple comparisons (0.05/8 = 0.00625). This analysis was provided for descriptive purposes, to understand which aspects of performance may have contributed to the overall performance difference between groups. As there was no simple performance difference between groups on the Implicit condition, not surprisingly there was no difference between groups found for any of the individual aspects of performance. On the Explicit condition, the children with autism completed fewer sets (*U* = 92, *p* = .005); no other comparisons were significant after Bonferroni correction was applied.

**Table 3:** Raw scores on measures of performance. Mann-Whitney U tests were used to test for differences between groups within each condition separately. Group differences were found only on the Explicit and not on the Implicit condition. Means are presented with standard deviations in parentheses. Asterisks (*) denote significant differences between groups at *p* < .00625.

	Non-autism (n = 25)	autism (n = 19)		Non-autism (n = 20)	autism (n = 19)	
	Implicit	Implicit	*U*	Explicit	Explicit	*U*
Sets completed	3.6 (3.25)	2.89 (2.71)	217.5	7.00 (2.45)*	3.53 (3.53)*	92.0
Trials to first set	13.56 (5.41)	15.22 (6.07)	213.0	10.55 (1.88)	12.28 (4.30)	137.0
Average trials per set	16.91 (4.13)	17.97 (3.82)	165.0	11.70 (2.5)	15.61 (4.36)	89.0
Average run length	7.04 (4.14)	7.23 (3.81)	211.0	9.59 (2.47)	17.38 (11.43)	122.0

In the next set of exploratory analyses, we examined error patterns. Table 4 shows the total errors, average errors per completed set, and types of errors committed by group and condition. Visual inspection of this data suggests that children with autism committed a high proportion of perseverative errors.

**Table 4:** Error patterns for children with and without autism. Total error counts, average errors per completed set, overall accuracy (proportion of all administered trials), as well as proportions of errors classified as perseverative, FMS, or random are provided. Proportions were calculated for each participant before aggregation, and thus may not sum to 1.0 (100%).

	non-autism		autism		non-autism		autism	
	Implicit		Implicit		Explicit		Explicit	
	Mean	SE	Mean	SE	Mean	SE	Mean	SE
Total errors	13.25	1.30	15.50	1.48	4.69	1.45	13.27	1.51
Average errors/ set	6.12	0.84	6.18	0.96	1.77	0.94	6.20	0.97
Overall accuracy	0.69	.038	.67	.04	.90	.04	.66	.04
Error Breakdown (proportion of total errors):								
Perseverative	0.44	0.06	0.51	0.07	0.19	0.06	0.54	0.07
FMS	0.20	0.05	0.20	0.06	0.23	0.06	0.22	0.06
Random	0.37	0.05	0.29	0.06	0.22	0.06	0.06	0.06

The total error and average errors were analysed using ANCOVA with WASI vocabulary and similarities scores as covariates. For total errors, significant main effects of both group, *F*(1,73) = 13.55, *p* < .001, and condition, *F*(1,73) = 14.17, *p* < .001, were found, as well as a significant interaction, *F*(1,73) = 4.96, *p* = .029. For average errors per completed set, significant main effects of both group, *F*(1,73) = 5.58, *p* = .021, and condition, *F*(1,73) = 5.45, *p* = .022, were found, as well as a significant interaction, *F*(1,73) = 5.67, *p* = .02. In both cases, the interaction is driven by lower error rates for children without autism in the Explicit condition.

Overall accuracy (inverse of error rate as a function of total trials administered) and the proportions of each error type were analyzed using logit model analysis with group and condition as fixed factors and WASI vocabulary and similarities raw scores as covariates. According to Jaeger [38] this method is preferred over arcsine transformations for analyzing proportions. For overall accuracy, main effects of both condition (Wald *Z* = -3.59, *p* < .01) and group (Wald *Z* = 11.32, *p* < .001), as well as a significant interaction were found (Wald *Z* = -8.56, *p* < .001); errors had an increased odds of 1.47 in the Implicit relative to the Explicit condition, and had an increased odds of 5.05 times among children with autism relative to the children without autism.

A three-factor Chi-squared test of independence was used to examine the distribution of errors by type, χ^2^(7) = 234, *p* < .001. As seen in Table 4, perseverative errors were the most prevalent type of error, with the exception of children without autism in the Explicit condition, where the rate dropped by more than half. FMS errors occurred at a consistent rate across group and condition (20–23%), and random errors occurred at a very low rate among children with autism in the Explicit condition. Based on the pattern of errors, it appears that children with and without autism were differentially affected by the experimental manipulation.

### Subgroup analysis

#### Subgroup 1 –“switchers”

In an attempt to understand the error patterns further, we examined switching by categorizing participants as switchers and non-switchers. We recoded the number of categories completed. Children who completed 0 or 1 category were labeled *non-switchers*, and those who scored 2–8 were labeled *switchers*. These non-switchers represent the most extreme inflexibility as they have failed the two-target level. In fact, the distribution of scores on the measure of total categories completed indicated that in the Explicit condition, children either passed or failed–if they could make one rule switch, they made them all. This was not the case in the Implicit condition, where all possible scores were represented for number of categories completed. Children with autism were significantly less likely than children without autism to switch rule sets altogether in the Explicit condition, (non-autism = 90% switched, autism = 42% switched), χ^2^ (1) = 10.01, *p* = .002, but not in the Implicit condition (non-autism = 64% switched, autism = 68% switched). Children classified as non-switchers would have a high number of errors classified as perseverative because there is no possibility of a random error on the two-target level and they did not make enough correct answers to score a FMS error (if they failed to maintain their incorrect set, it would be marked as correct). When we removed the “non-switchers” and re-examined the errors, main effects of both condition (Wald Z = -7.91, p < .01) and group (Wald Z = 3.53, p < .001) remained with no significant interaction; errors had an increased odds of 4.22 in the Implicit relative to the Explicit condition, and had an increased odds of 2.14 among children with autism relative to children without autism. The Chi-squared test for independence for the distribution of errors by type was also significant (χ^2^(7) = 38.28, p < .001). Perseverative errors remained the most frequent error type for autism, 69% and 50% in the Explicit and Implicit conditions respectively (Table 5). Children without autism only committed more perseverative errors in the Explicit condition, 39% and 35% in the Explicit and Implicit conditions respectively; in the Implicit condition they committed more random errors (46%).

**Table 5:** Error breakdown (type of error as a proportion of all errors) and overall accuracy for “switchers” only.

	Non-autism (n = 17)	autism (n = 13)	Non-autism (n = 18)	autism (n = 8)
	Implicit	Implicit	Explicit	Explicit
Overall Accuracy	0.87	0.76	0.97	0.94
Perseverative	0.35	0.50	0.39	0.69
FMS	0.20	0.10	0.31	0.21
Random	0.46	0.40	0.31	0.10

We also re-examined simple performance in this subgroup of switchers and found only a main effect of condition, *F*(1,48) = 56.49, p < .01, partial η^2^ = .541. Both groups performed better in the Explicit condition than the Implicit condition, with gains of 103–114 points. Table 6 presents the means and standard deviations of each aspect of performance.

**Table 6:** Mean raw scores on measures of performance for “switchers” only. Standard deviations are given in parentheses.

	Non-autism (n = 17)	autism (n = 13)	Non-autism (n = 18)	autism (n = 8)
	Implicit	Implicit	Explicit	Explicit
Sets completed	4.94 (2.83)	4.08 (2.46)	7.67 (1.41)	7.25 (2.12)
Trials to first set	10.75 (.86)	12.75 (4.58)	10.44 (1.89)	10.63 (1.77)
Average trials per set	14.74 (2.10)	15.73 (1.81)	11.02 (1.46)	11.63 (2.13)
Average run length	7.13 (1.65)	6.87 (2.56)	9.28 (1.44)	9.45 (1.16)

#### Subgroup 2 –non-switchers

Removing the non-switchers also resulted in severely unbalanced groups with sample sizes ranging from 8 to 18, and similar examinations of the non-switchers (with sample sizes ranging from 2 to 11) failed Levene’s test of equality of error variances and could not be examined with ANOVA. Based on a visual examination of stem-and-leaf plots, there did not appear to be any group or condition differences in simple performance among the non-switchers. Descriptively, the non-switchers in both groups committed a higher proportion of perseverative errors. This pattern was most pronounced in the Explicit condition, with perseverative errors accounting for 85% (non-autism) and 91% (autism) of errors. Given that participants began with the two-target level and by definition the non-switchers did not progress beyond that level, non-switchers had limited opportunity to commit non-perseverative errors.

The non-switchers’ performance was further examined for differences in passing or failing the “pre-switch” of the two-target level. In the Explicit condition, 2/11 children with autism failed pre-switch, compared to 0/2 children without autism. In the Implicit condition, 4/6 children with autism failed pre-switch, compared to 5/8 children without autism. While these numbers are not appropriate for analysis, it appears that most children with autism and all children without autism could successfully sort one dimension to criterion in the Explicit condition, but for both groups this was more challenging in the Implicit condition.

### Predictive modeling of task performance

**Fig 4:**
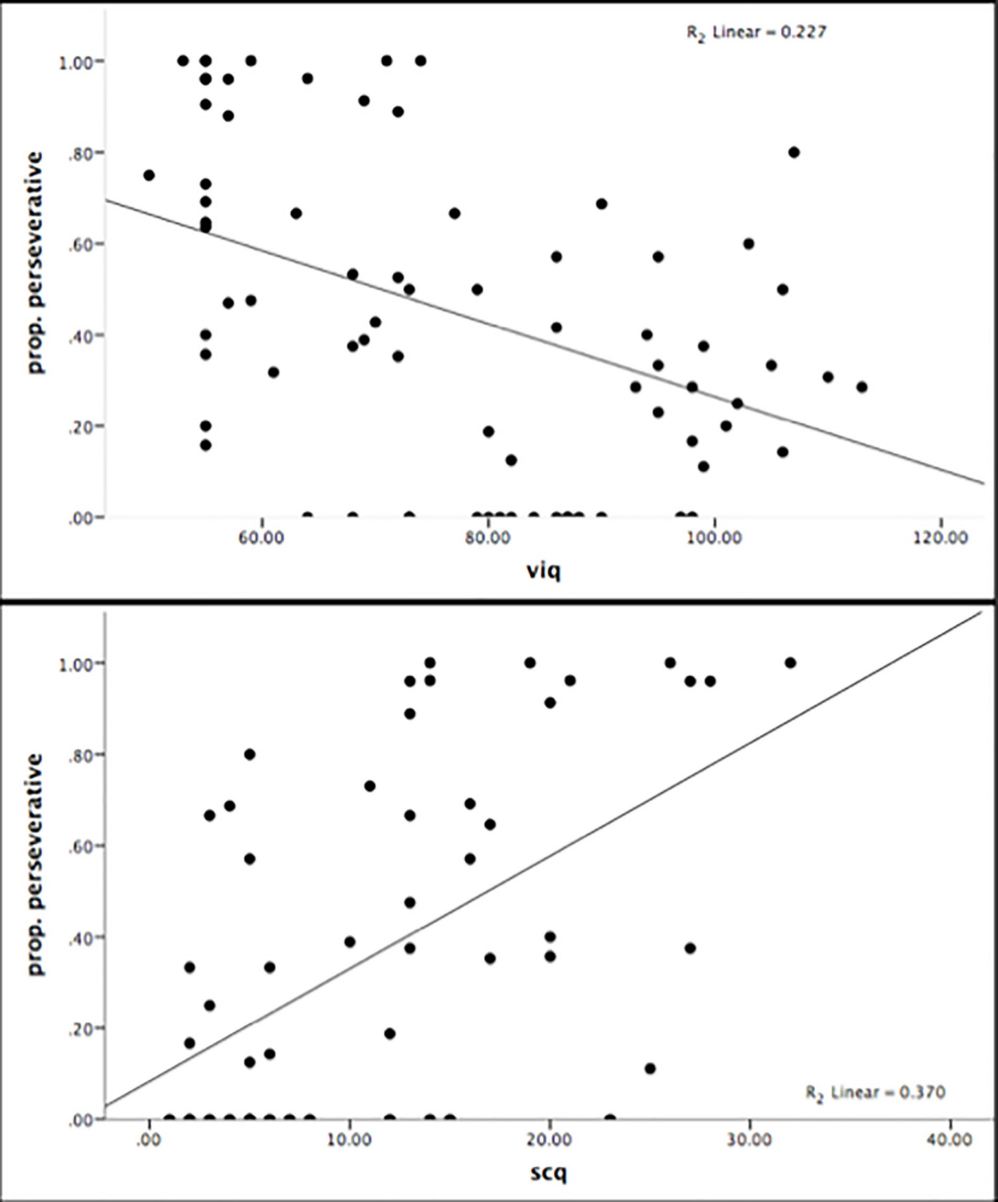
Scatterplot of the relationship between proportion of perseverative errors and VIQ (top) and SCQ (bottom).

Rather than dismiss the non-switchers as failing the task, we used logistic regression to model rule-switching. We initially tested a model that included Condition, however this factor was not a significant predictor and data for the two conditions were considered together. We used a forward conditional model and entered VIQ, PIQ, SCQ scores, and age as predictors; SCQ scores were the only significant predictor (*β* = -0.14, *p* = .006) in the model, which correctly classified 80.4% of participants, *R*^2 =^ .25. We also used linear regression to model number of categories completed and proportion of errors classified as perseverative separately with SCQ, VIQ, PIQ, and age as predictors. Verbal IQ significantly predicted the total number of sets completed (*β* = 0.09, *p* < .001, *R*^2 =^ .23) with no significant contribution from the other predictors, whereas VIQ significantly predicted the proportion of errors classified as perseverative (*β* = -0.01, *p* < .001, *R*^2 =^ .32) with an additional 6% of variance contributed by SCQ (*β* = 0.01, *p* = .047). Thus, individuals with higher SCQ scores, regardless of IQ, were less likely to switch rule sets; VIQ uniquely contributed to completing more sets but VIQ and SCQ together predicted perseverative errors (Fig 4). As non-switchers score very low on total sets completed and very high on perseverative errors, it appears these regression analyses are identifying the moderate perseverators; participants who are not so symptomatic that they cannot switch, nevertheless continue to be plagued by moderate perseveration. For these moderate perseverators, higher VIQ and lower symptomology could be relevant to completing more sets and to resisting perseveration.

## Discussion

The WCST is a widely used test of cognitive flexibility and numerous studies find that performance on the WCST, and cognitive flexibility in general, is a challenge in autism[2]. The aim of this study was to examine cognitive inflexibility by manipulating the rule structure; we presented children with a card-sorting task that increased in the number of potential rules; in addition, we randomly assigned children to one of two instruction conditions. The explicit condition instructions were based on the pre-school DCCS, and the implicit condition instructions were based on the WCST. While children without autism performed better overall when the rule structure was explicit than when rules had to be self-generated, the participants with autism benefitted less. The participants with autism committed more errors on both conditions, and both groups committed more perseverative errors when the rule structure was explicit. More than half of participants with autism (58%) performed at a level consistent with typically developing 3-year-olds[3, 4]. In contrast, only 10% of participants without autism performed at this level. This was not seen in the Implicit condition, a finding that cannot be explained with reference to chance differences in the basic ability level of different groups given that participants were randomly assigned to conditions.

The finding of increased errors, and particularly increased perseverative errors among participants with autism is consistent with previous research [2, 12–19]. The finding of enhanced performance in the Explicit condition relative to the Implicit condition is not unexpected, however performance of the participants with autism in the Explicit condition was surprising.

On examining the composite score of simple performance, which took into account five measures of performance accuracy and efficiency, it seems the Explicit condition improved card-sorting performance in both children with and without autism, although children with autism did not demonstrate as large an improvement as children without autism. Hence, the Explicit condition was easier.

For a subset of children with autism, the Explicit provision of rules appears to have increased perseveration, and a large proportion of children with autism in this condition were unable to switch sets even once. As participants were randomly assigned to and did not differ on cognitive level between the two conditions, it seems the manipulation of task instructions caused the increase in perseveration such that the number of children with autism completely unable to switch nearly doubled, from 6/19 to 11/19.

One potential explanation is that the need to self-generate a sorting rule reduced the arbitrariness of the rules for children with autism. According to Russell and colleagues [39, 40], cognitive flexibility is especially poor in autism when the rules appear arbitrary, for example on the Windows Task, where children have to inhibit reaching directly into the box and instead perform an arbitrary action in order to retrieve a candy. When the rules are logical, children with autism perform better. In this task, because the feedback of the Implicit condition forced children to generate the rule, perhaps the rules effectively seemed less arbitrary than when they were asserted by the experimenter.

While these findings were unexpected, there is reason to believe that typically developing 3-year-olds might perform similarly to the children with autism, despite our findings that the Explicit condition was much easier among typically developing children aged 6 years and older. Like children with autism, typically developing 3-year-olds struggle with cognitive flexibility. Among 3-year-olds, feedback is reported to improve the ability to inhibit pre-potent responses on both the Windows task [41] and the Marbles task [42]. Feedback also improved performance among typically developing children when coupled with Explicit instruction on the DCCS [43]. A meta-analysis of DCCS performance in 3-year-olds found the most support for inhibitory control models of performance [3]; a given sorting rule has been activated, and owing to age-related immaturity of neural development, the 3-year-old cannot inhibit rule A in favour of conflicting rule B. Future studies will be needed to examine how typically developing children 3 year olds would perform on this task. We did not include pre-schoolers in this study as the children with autism in our sample were of school-age and ability. We also did not include a third condition in which participants with autism were presented with both the Explicit instruction and given corrective feedback. Like typically developing 3-year-olds, we might expect such a condition to enhance performance for participants with autism.

While 45% of participants with autism were “non-switchers,” amongst the switchers, the Explicit condition improved performance on card-sorting. When the non-switchers were removed, the group differences on the Explicit condition disappeared and both groups evidenced improved performance and decreased perseveration in the Explicit condition, although children with autism continued to commit more errors and disproportionately more perseverative errors than children without autism. Thus, the explicit provision of rules influenced performance differently, depending on whether children with autism could be classified as non-switchers or switchers. This classification was predicted by severity of autism symptoms, not general measures of IQ, while verbal IQ helped predict perseveration rates, which remained elevated among switchers as well. The importance of symptomology as a predictor may shed light on issues of heterogeneity reported in the literature; non-switchers had higher SCQ scores than switchers. Landry and Al-Taie [2] examined predictors of WCST performance and found symptomology (ADI scores) was associated with perseveration, however chronological age was a confound in this relationship. Further, they found performance IQ, not verbal IQ, was associated with perseveration; verbal IQ predicted non-perseverative errors.

Our regression modeling indicated that SCQ scores, a measure of symptomology, predicted which children would and would not switch sets and neither VIQ nor PIQ contributed additionally to the model. This suggests cognitive inflexibility is intimately associated with the outwardly visible symptoms of autism; the more “autistic-like” behaviours reported by parents, the less likely they were to switch sets. This was also seen in the children without autism who were excluded on the basis of excessively high SCQ, all of whom failed to switch. While it might be tempting to take this as evidence that the social nature of the task administration creates a disadvantage for participants with autism, the notion that individuals with autism perform better on computerized versions of the same task has been disputed [2].

### Limitations and future directions

We did not in this study examine the psychometric properties of our task. This task was designed as an experimental manipulation, and is only intended for between condition and between group comparisons. Further research examining the relationship between this task and other executive function tasks, both lab-based and “real-world” would be needed before any assertions about clinical evaluation of executive function could be made. We did not record the gender of our participants at the time of testing, and thus we are unable to examine whether there were any gender differences within and across groups on this task. We also did not collect any measure of working memory, and thus are unable to assess the degree to which individual differences in verbal working memory may impact on task performance in the two conditions.

We recruited and tested our participants within the school setting. We are confident in the diagnostic classifications and parent/teacher confirmation that no participants were diagnosed with ADHD. The SCQ is a screening measure, not a diagnostic measure. We are confident in the diagnostic classification of our participants with and without autism and chose the SCQ to give an indication of symptomology that would have enough variability in both groups to be a useful measure. Diagnostic tests such as the ADOS [44] or ADI [37], while rigorous for clinical purposes, do not give a very wide range of scores and do not capture variability within or outside the autism spectrum. Future studies should examine relationships between cognitive flexibility and other continuous measures of symptomology such as the Social Responsiveness Scale [45], which offers a continuum of scores on an even larger scale, and capacity to examine as a function of subscales relevant to the autism spectrum.

Our autism and non-autism groups differed significantly on chronological age, however they were matched for developmental level, not chronological age. As highlighted by Burack et al. [46], “no choice of comparison group or matching strategy is perfect, but rather needs to be determined by specific research objectives and theoretical questions” (p. 65). In this study, we based our matching strategy on the rationale that verbal skill development is relevant to performance on the experimental task, as described in the Introduction. The participants with autism as a group were older than the non-autism group, yet this afforded them no advantage as seen in their performance. For a more detailed discussion of issues in matching strategies, please see [46, 47].

Many behaviours characteristic of autism vary within the normal population; the robust relationship between SCQ and switching suggests cognitive flexibility may be one such behaviour. While SCQ was a robust predictor of switching, participants were randomly assigned to the Explicit and Implicit conditions, and there was no evidence to suggest SCQ scores differed (*p* > .8) between conditions, yet children with autism in the Explicit condition were more likely to be non-switchers and perseverated more. We analysed the performance of all participants, regardless of switching status. The rationale for this decision is that we were interested in many aspects of performance, not just switching. Oftentimes we discard participants who are unable to perform a task. Sometimes this is legitimate, but when a substantial number of participants fail, and they are disproportionately in one condition, this warrants investigation. Why did more than half of children with autism effectively fail the Explicit condition, when only 10% of the children without autism failed, and only 1/3 of both groups failed in the harder Implicit condition? Further research is needed to examine why the Explicit instructions were so handicapping to the children with autism, and how symptom severity relates to that effect. Should these findings be replicated using a within-subjects design, we would have even more compelling evidence that the instruction alone could have such a detrimental effect on flexibility. Presenting the two conditions to the same group of participants carries a unique set of challenges. Presumably there would be carry-over effects, which might differ between children with autism and a comparison group, further muddying interpretation.

In conclusion, we replicated the finding of increased perseveration among participants with autism, and found the experimental manipulation differentially affected participants with and without autism. What are the possible explanations? We reject the notion that cognitive flexibility can account for the differential performance on the Explicit condition because cognitive flexibility demands were equal in the Implicit condition. We can also reject a working memory explanation because working memory demands would be higher in the Implicit condition. The two experimental conditions differed in instructions and in the feedback provided. When children with autism were provided with explicit sorting instruction and no corrective feedback, 42% of children were successful at switching sorting rules. When children with autism were not provided with the sorting rule and had to work it out on the basis of corrective feedback, 68% were successful at switching sorting rules. We can only speculate as to how corrective feedback would have impacted performance in the Explicit condition, as this has been shown to improve performance for typically developing children on the DCCS, however the original DCCS does not include corrective feedback for children. The differential performance of children with autism on our condition inspired by the DCCS and our condition inspired by the WCST suggests that the tasks are less analogous than previously thought.

## Supporting information

S1 File List of target cards used in each level of the card sort task. (DOCX)

S2 File Instructions given to participants in the Explicit and Implicit conditions. (DOCX)
