# Supplementary material for: An examination of perseverative errors and cognitive flexibility in autism
Source: PLoS One. 2021 Jan 13;16(1):e0223160. doi: 10.1371/journal.pone.0223160 (PMC7806145; doi:10.1371/journal.pone.0223160)
Supplement: S1 File — (DOCX) [file pone.0223160.s001.docx]

Two-target level

yellow stars

red squares

Three-target level

one green square

one yellow circle

two green stars

two red circles

three green stars

three red squares

Four-target level

one blue square

one blue circle

one yellow triangle

one green triangle

two blue stars

two blue circles

two red triangles

two green triangles

three blue stars

three blue squares

three red triangles

three yellow triangles

four red squares

four red circles

four yellow stars

four yellow circles

four green stars

four green squares

(plus all the cards in the three-target level)
