# Supplementary material for: An examination of perseverative errors and cognitive flexibility in autism
Source: PLoS One. 2021 Jan 13;16(1):e0223160. doi: 10.1371/journal.pone.0223160 (PMC7806145; doi:10.1371/journal.pone.0223160)
Supplement: S2 File — (DOCX) [file pone.0223160.s002.docx]

Supplementary File 2: *Instructions given to participants in the Explicit and Implicit conditions.*

## All participants were told:

“We are going to play a special card game. See here we have a red star and a yellow square. On these cards we have yellow stars and red squares. We could put the star with the star (experimenter demonstrates) and the square with the square, because they are the same shape. Or, we could put the red one with the red one and the yellow one with the yellow one because they are the same colour. The way we play the game is that I am going to give you one card at a time, and I want you to put it face down where you think it should go.”

Participants in the Explicit condition were told:

“I will tell you how I want you to put the cards. Listen carefully because the way I tell you to put the cards might change.”

Participants in the Implicit condition were told:

“You have to figure out what is the right way to put the cards. After you have placed the card, then I will tell you if it was right or if it was wrong. Listen carefully because the right way to put the cards might change.”
